# Recommendations for the COVID-19 Response at the National Level Based on Lessons Learned from the Ebola Virus Disease Outbreak in the Democratic Republic of the Congo

**DOI:** 10.4269/ajtmh.20-0256

**Published:** 2020-05-19

**Authors:** Linda Meta Mobula, Hadia Samaha, Michel Yao, Abdou Salam Gueye, Boubacar Diallo, Chantal Umutoni, Julienne Anoko, Jean-Pierre Lokonga, Luigi Minikulu, Mathias Mossoko, Emanuele Bruni, Simone Carter, Thibaut Jombart, Ibrahima Soce Fall, Steve Ahuka-Mundeke

**Affiliations:** 1 Ebola Response Team, Health, Nutrition and Population, Global Practice, World Bank Group, Washington, District of Columbia;; 2 Center for Humanitarian Health, Johns Hopkins School of Public Health, Baltimore, Maryland;; ^3^Division of General. Internal Medicine, Department of Medicine, Johns Hopkins School of Medicine, Baltimore, Maryland;; 4 World Health Organization, AFRO, Health Emergencies Programme, Brazzaville, Democratic Republic of the Congo;; 5 World Health Organization, Country Office, Nouakchott, Mauritania;; 6 Ebola Response Team, UNICEF, Goma, Democratic Republic of the Congo;; 7 Programme de Développement du Système de Santé, Ministry of Health, Goma, Democratic Republic of the Congo;; 8 Ebola Technical Secretariat, Goma, Democratic Republic of the Congo;; 9 World Health Organization, Health Emergencies Programme, Geneva Switzerland;; 10 London School of Tropical Medicine and Hygiene, London, United Kingdom

## Abstract

The tenth outbreak of Ebola virus disease (EVD) in North Kivu, the Democratic Republic of the Congo (DRC), was declared 8 days after the end of the ninth EVD outbreak, in the Equateur Province on August 1, 2018. With a total of 3,461 confirmed and probable cases, the North Kivu outbreak was the second largest outbreak after that in West Africa in 2014–2016, and the largest observed in the DRC. This outbreak was difficult to control because of multiple challenges, including armed conflict, population displacement, movement of contacts, community mistrust, and high population density. It took more than 21 months to control the outbreak, with critical innovations and systems put into place. We describe systems that were put into place during the EVD response in the DRC that can be leveraged for the response to the current COVID-19 global pandemic.

## INTRODUCTION

On August 1, 2018, the Democratic Republic of the Congo (DRC) declared its tenth outbreak of Ebola virus disease (EVD) in North Kivu. This outbreak was declared 8 days after the end of the ninth EVD outbreak in the Equateur Province, with a total of 54 confirmed and probable cases.^1^ With a total of 3,453 confirmed and probable cases, the North Kivu outbreak remains the second largest EVD outbreak, after the West Africa outbreak in 2014–2016.^2^ Several security incidents, including attacks by armed groups, hindered implementation of activities by causing a reduced operational presence, rendered certain health zones inaccessible, and likely led to increased transmission.^3,4^ Since January 2019, more than 350 incidents that disrupted response activities were recorded, 80% of which were directly targeted at structures or response personnel.^4^ Population displacement, conflict, a highly dense population, mistrust, and insufficient aid for basic services impacted response activities.

The EVD response was government led with the support of a number of technical and strategic partners such as the WHO, UNICEF, the World Food Program, the International Organization of Migration (IOM), the U.S. CDC, the UN Office for the Coordination of Humanitarian Affairs, and a number of nongovernmental organizations. Donors worked in concerted and harmonized ways to support the response. The response was driven by a unique National Ebola Strategic Response Plan with a unique budget. The public health response was coordinated by the Multi-sectoral Technical Secretariat and the Ministry of Health. Core pillars of the response were coled by the government and technical partners such as the WHO, UNICEF, the IOM, and the International Federation of the Red Cross (see Table 1).

**Table 1:** Public health response pillars (technical areas)

Sub-pillar	Lead	Colead
1. Risk communication and community engagement	Ministry of Health	UNICEF
2. Surveillance, contact tracing, and vaccination	Ministry of Health	WHO
3. Laboratory and research	Ministry of Health	WHO
4. Case management, free care, and survivor care	Ministry of Health	WHO
5. Infection, prevention, and control/water, sanitation, and hygiene	Ministry of Health	WHO/UNICEF
6. Safe and dignified burials	Ministry of Health	International Federation of the Red Cross
7. Psychosocial support	Ministry of Health	UNICEF
8. Operational preparedness	Ministry of Health	WHO
9. Coordination	Ministry of Health	
10. Support to coordination	Ministry of Health	World Bank, WHO, and UN Office for the Coordination of Humanitarian Affairs

The main strategic objective of the EVD response was to break chains of transmission, by ensuring rapid detection and isolation of cases, intensification of multidisciplinary public health measures around a confirmed case, strengthening of community engagement activities, strengthening of health systems, and effective coordination of both local and international partners.

On December 31, 2019, the WHO China Country Office was informed of cases of pneumonia of unknown etiology detected in Wuhan city, Hubei Province. By January 7, 2020, Chinese scientists isolated a novel coronavirus.^5^ The COVID-19 outbreak was declared a public health emergency of international concern on January 30, 2020. On March 11, the WHO declared a global pandemic of COVID-19 as a result of the spread of SARS-CoV-2.^6^

## LESSONS LEARNED FROM THE DRC 10th EVD OUTBREAK

Although modes of transmission and strategies to “flatten the curve” differ between EVD and COVID-19, there are broad lessons learned from the EVD response that can generally be applied to the COVID-19 response. An effective COVID-19 response focuses on rapid detection via testing, contact tracing, isolation, treatment, and mitigation measures including physical distancing. We describe critical systems that were put into place during the tenth EVD response in the DRC that we believe can be leveraged for the response to the current COVID-19 pandemic (Table 2).

**Table 2:** Key interventions to consider for the COVID-19 response

Technical area	10th Democratic Republic of the Congo Ebola virus disease response	Relevance for COVID-19 response
Response coordination	Incident management system to improve information flow and decentralize decision-making	Use of an incident management system to support coordination of COVID-19 response
		Under government leadership, ensure routine strategic meetings
	Creation of functional groups with clear roles and responsibilities to ensure improved span of control and chain of command	Need for a multi-sectorial response
	Decentralized operational coordination at the subdistrict level	Development of key performance indicators to ensure corrective action for critical response interventions
	Monitoring framework comprehensive: inputs, outputs, outcomes, and impacts	
	Key performance indicator developed to ensure corrective action	
Surveillance	Monitoring unit established to improve follow-up of lost contacts	Establish monitoring uni/structure to improve follow-up of contacts lost to follow-up
		Involve community structures at early stage of surveillance activities to generate alerts
	Food distribution provided to contacts
		Consider food distributions to communities under isolation/quarantine
	Community leaders involved in ensuring proper contact tracin	
		Identify individuals with field epidemiology expertise to conduct surveillance activities (including contact tracing)
		Conduct rapid training of surveillance team members to investigate alerts, and collect and analyze epidemiologic information
	Active case finding and door-to-door activities implemented to improve case detection coupled with community watch interventions to ensure tracking of movements (new arrivals, deaths, and illnesses)	
		Investigate alerts reported by households, community leaders, or health facilities and report validated alerts within 24 hours
	Active case search in health facilities	
	Functional triage systems in health facilities	
	An alert monitoring and investigation platform that helped investigate cases within 24 hours	
Risk communication and community engagement	Community-centered approach with feedback mechanisms to follow and address rumors	Early involvement of anthropologists and social scientists in the development of risk communication and community engagement approaches
	Anthropologists and social scientist engaged to provide feedback on different response measures	
		Creation of feedback mechanisms to better target activities
	Trust gained from local religious, traditional, and community leaders to mitigate community reticence	
		Prepare communities to play active role with other response interventions
	Community structures and community health workers who speak local language used to better communicate with communities	
		Early identification and engagement with community leaders to mitigate community reticence to response interventions
	Anthropologists and/or social scientists included in part of the response	
Infection prevention and control (IPC)	Established standardized package for IPC	Define and implement a standardized IPC package
		Capitalize on IPC tool kit and standard package for training of trainers
	Implemented ring IPC with supervision (IPC focal point at health facilities) and frequent evaluations (use of IPC score card)	
		Target traditional healers and pharmacists
	Used evidence to adapt and improve strategy	
Case management and free care	Decentralized transit centers used to rapidly test and isolate cases in setting close to communities, which also improved willingness to seek care	Consider a similar model of decentralized care and testing
		Disseminate standardized guidelines on optimized care based on existing/evolving evidence
		Ensure that free-care models can cope with increased use of health services
	Create SOPs and guidelines for optimized care based on existing evidence	
		Consider compassionate use for investigational drugs and conduct studies to look at effectiveness
Operational preparedness	Defined a package of activities for operational preparedness to reduce the risk of spreading Ebola virus disease to at-risk areas	Anticipate mechanism to increase capacity for control measures (early detection, investigation, laboratory confirmation, isolation, and treatment
	Deployed experts to at-risk health zone to implement readiness activities and strengthen the health system	Work on mass training mechanism and prepositioning of treatment items (critical care, ventilators…)
		Conduct training to equip health zones based on clear protocols and package of activities
		Use similar preparedness package of interventions for COVID-19
	Trained rapid response teams to investigate alerts in non-affected health zones	
Analytics cell	Set up epidemiological and social sciences analysis structure to provide real-time integrated analysis	Develop integrated analysis structure to provide real-time insights and design appropriate response
		Monitor epidemiolocal trends beyond that of the outbreak (concurrent diseases) to mitigated impacts of outbreak and response
		Monitor perceptions and reported use of health services
	Regularly monitoring and understanding of health behavior trends (perceptions and reported use—mixed analysis)	
	Used evidence to inform different response measures	
	Set up mechanisms to monitor and track recommendations	
Donor coordination	Preparation of a unique strategic response plan, with validated unit costs for all response interventions	Ensure global donor coordination
		Ensure alignment with national strategies
		Establish processes, including eligibility criteria for hazard payments, pay scales, and payment modalities, as well as mechanisms to systematically list healthcare workers
	Involvement in the planning process and continuous interaction to share challenges and gaps to be filled	
	Ensuring resources as well as technical support were provided just in time based on priority areas and gap filling.	

### Coordination.

Coordination of international and national counterparts during a public health emergency is critical, with an accountability system and clear understanding of who is in charge. Coordination for the EVD response was under the leadership of the MOH and Technical Secretariat, a multisectoral body put into place by the presidency. Clear roles for the coleads (United Nations and other international organizations) were established. Command and control of operations was centralized in Goma, with decentralized sub-coordination at the health zone level.

The lack of standardized data on the implementation of operations during the West African EVD outbreak led to the creation of a monitoring framework. This provided operational and strategic analyses to enable partners and donors to follow-up on response outcomes. The monitoring framework in the DRC represented one of the first attempts to use a harmonized, multisectorial, and real-time monitoring system that maintained situational awareness to evaluate short- and medium-term impacts of activities.^7^

The Technical Secretariat adopted an incident management system, whose aim was to improve information flow and decentralize decision-making. It created clear roles and responsibilities to ensure improved span of control and chain of command, which was necessary because of the lack of prompt decision-making at the coordination level.

For COVID-19, national governments should consider using an incident management system to support coordination of the response at the national level. This will allow for decentralized and prompt decision-making on operations and provision of technical guidance. Under the leadership of the government, strategic meetings should be held regularly, with lead agencies involved in the operationalization and technical leadership of the response. Countries should consider adopting a monitoring framework that looks at performance indicators to guide the implementation of multi-sectorial operations in real time.

### Surveillance.

Public health performance indicators at the beginning of the EVD response were poor, with increasing community deaths, poor contact tracing (indicated by the high number of cases that had no known contacts), and delays between symptom onset and isolation. A decline in cases toward the end of 2019 was thought to be due to improvement in the quality of surveillance activities, including prompt investigation, early detection and isolation of cases, an adaptive vaccination strategy (from ring strategy to geographic vaccination), community-based surveillance, and the prioritization of follow-up with high-risk contacts. Food distribution was provided to contacts to restrict their movement, a practice begun in West Africa by the World Food Program.

Having a platform of rapid response teams, the use of innovations such as Go.Data, a software developed by the WHO and Global Outbreak Alert and Response Network partners, enhanced the monitoring of contact tracing data in the DRC, including the visualization of chains of transmission and contact follow-up. Community health workers were trained to conduct contact tracing, with a strong supervisory system, to ensure quality interventions.

As with EVD, it is critical that for the COVID-19 response, community structures and leaders be involved at an early stage in surveillance activities. Case isolation and aggressive testing, and involvement of trusted community leaders in the promotion of these interventions, can help coordinate and improve the effectiveness of control measures. Rapid training should be conducted for surveillance teams to be able to investigate alerts, investigate cases and clusters early in the outbreak, conduct contact tracing within 24 hours, collect and analyze epidemiologic information, and adjust messaging appropriately.

Community-based surveillance and alert systems linked to a prompt investigation system can help to address contact tracing challenges for COVID-19 in many countries. It is important that community structures be involved at an early stage in surveillance activities, and community leaders should be involved in ensuring proper contact tracing.

Similar software systems as for EVD should be considered for contact tracing for COVID-19. In addition, the use of community volunteers can help create a robust workforce for contact-tracing activities. Those with field epidemiology or community health outreach should be identified early and can be leveraged for contact-tracing activities.

Other innovations, such as a dedicated unit/structure established for the follow-up of lost contacts and never seen contacts (as was done for EVD), can be put into place to ensure improved contact tracing. COVID-19 strategies at the national level often require quarantine and isolation, which is difficult to do in low- and middle-income countries. The distribution of food to those that are experiencing food insecurity becomes critical. It may help to restrict movement of contacts and enforce compliance to confinement measures.

### Analysis cell.

Dedicated to analyzing response-related data, the social sciences and epidemiological analysis cell was established to provide critical and timely assessment of the epidemiological situation and to inform EVD response activities. The systematic use of both statistical epidemiology and social sciences data provided a comprehensive understanding of context, with epidemiological analysis identifying new patterns in the data and social sciences providing explanations on underlying causes. In 2019, the implementation of novel outbreak analytics approaches permitted further developments of both routine monitoring activities and operational research, thereby increasing capacity for in-depth analyses. This allowed for enhanced situational awareness over the course of the epidemic, leading to identification of aspects of the response needing strengthening, and generally improving surveillance. The social science cell also set up an online recommendation tracking tool, to monitor actions by study, by response measure and by location over time. Using this tracking mechanism enabled response teams to evaluate effectiveness of response interventions and monitor impact using evidence.

For COVID-19, to have real-time data that inform the response, governments should consider establishing an analytical cell, embedded within the response structure, which would enable improved understanding of the epidemiological situation as well as community perception and practice, allowing for evidence-based targeted programming that continuously informs the national strategy.

### Risk communication and community engagement.

In the DRC, existing local community structures were not included at the beginning of the EVD response. Rather, the inclusion of these structures from the outset should be the cornerstone of all community engagement activities.

The inclusion of traditional, religious, and political leaders was delayed in the DRC, and the use of appropriate language was not included early. Engagement with trusted community, political, and religious leaders offers an essential gateway to the community. Early identification and engagement of community leaders is essential to achieve acceptance and compliance with public health measures, as these leaders are critical points of access to their communities.

The Democratic Republic of the Congo has five national languages and more than 300 local languages. Therefore, utilization of the appropriate local language by community mobilizers, who were recruited from their own communities, was critical. For example, Kinande and Kiswahili are spoken by the Nande population that was predominantly affected by the EVD outbreak. Instead, many organizations used French and Lingala (a lingua franca used by the military), which are national languages, making it challenging for the Nande and other communities to comprehend health messaging.

In the DRC, a community-centered approach was adopted, with feedback mechanisms to follow and address rumors and to inform other response pillars (such as surveillance, infection prevention and control (IPC), and safe and dignified burials). Very early on, risk communication anthropologists were engaged to support different pillars on addressing and mitigating community tensions and reticence, as well as to work with feedback mechanisms, ensuring proper communication.

For COVID-19, the early involvement of trusted leaders is critical in ensuring that communities adhere to public health measures, such as physical distancing practices. For COVID-19, the use of local languages for all risk communication and community engagement activities is key. In addition, COVID-19 national responses should include early involvement of anthropologists and social scientists to target behavioral change around mitigation measures such as social distancing. This should be coupled with the creation of feedback mechanisms from communities to better target activities, and community-based surveillance and sensitization for preventive measures. The ability to incorporate a greater understanding of the perceptions and practice of communities and healthcare workers is likely to result in the development of more targeted programming.

### Infection prevention and control.

Nosocomial transmission played an important role in the spread of EVD, with 5% of infections occurring among healthcare workers and high incidence of nosocomial infections within private and traditional health centers. A standardized package for IPC/water, sanitation, and hygiene was established to ensure a coordinated IPC strategy. Supervision (establishing an IPC focal point at health facilities) and frequent evaluations (use of an IPC score card) were put into place. Evaluations helped in developing plans to fill gaps and monitor response progress. Traditional healers and pharmacists were involved in IPC training, albeit late, as they played an important role in the spread of Ebola. Triage systems set up in health facilities helped to ensure health service continuity, allowing access to health services for regular health care. Applying IPC measures allowed mass malaria drug administration and a measles vaccination campaign.

For COVID-19, given the high number of healthcare providers that are becoming infected, an evaluation of the IPC systems at health facilities that informs planning remains critical. Monitoring of programming should be put in place as soon as possible, involving all the stakeholders.

### Case management and free care.

In the EVD response, protocols for optimized care were established and disseminated. The monitored emergency use of unregistered and investigational interventions for EVD trial was put into place early, allowing for the use of therapeutics under compassionate care for confirmed EVD patients. Despite the challenging security environment, a randomized controlled trial testing the efficacy of investigational drugs was quickly put into place.^8^ Decentralized transit centers with the ability to rapidly conduct testing and isolation close to communities were critical in improving access to care.

COVID-19 has many similarities to EVD in that there is not a known cure. There is a need for the creation of treatment protocols describing optimized care that will be rapidly updated with evolving evidence. The use of investigational drugs under compassionate care should be done on a case-by-case basis for COVID-19, with clear guidelines for their use, and collection of data on their effectiveness. Countries should be advised to facilitate rapid ethical clearance processes for clinical trials examining the effectiveness of investigational therapies.

To prevent increased morbidity and mortality from other diseases, mechanisms should be in place to ensure continuity of standard health care, including a triage mechanism and a system for staff repurposing and maintenance of supply chains for essential medicines.

### Operational preparedness.

In the EVD response, a package of activities was defined for operational preparedness to reduce the risk of spread to at-risk health zones. Experts were deployed to implement readiness activities. Rapid response teams were trained to investigate alerts in non-affected health zones, providers were trained in standard IPC measures, mentorship was organized in health centers, and isolation rooms were identified and equipped in health facilities. Preparedness measures allowed for a prompt response in new provinces and cities, even in the neighboring country of Uganda.

For COVID-19, preparedness based on a readiness assessment should lead to activities to ensure rapid detection of cases and a comprehensive response. This should include developing preparedness and response plans, training of response teams, prepositioning of supplies, and preparing treatment centers. Preparedness activities should be decentralized as quickly as possible to be ahead of the spread of the disease.

### Multi-sectoral approach.

Challenges such as limited access to potable water, food insecurity, and armed conflict all contributed to the difficulty in containing the EVD outbreak. There was a need to ensure the continuity of services, to mitigate potential secondary impacts of the outbreak, and to deliver response interventions. There was also an important need to consider community needs in addition to the EVD outbreak. Responding to what communities perceive as their priorities is key for community engagement and acceptance.

For COVID-19, there is a need for a multi-sectorial approach, enabling interventions to increase compliance to public health measures and avoid secondary problems such as disease outbreaks, food insecurity, and economic hardship.

### Donor coordination, accountability, and transparency.

The tenth EVD outbreak resulted in mobilizing important funding to support strategic plans. Aligning and harmonizing engagement with the national government was instrumental for donors to ensure that resources and technical support were provided based on priority areas and gap filling. To ensure greater accountability and transparency, a fiduciary system was put in place. The main objective was to provide government, implementing partners, and donors relevant information required to ensure adequate and timely flow of funds and make informed strategic, technical, and operational decisions.

For COVID-19, financing of a response should include the preparation of a unique strategic response plan, with validated unit costs for all response interventions. Based on lessons learned from EVD, it is critical to establish processes, including eligibility criteria for hazard payments, pay scales, mechanisms to systematically list healthcare workers, and payment modalities.

## CONCLUSION

Key lessons learned from the 10th DRC EVD outbreak response ought to be implemented as key interventions in the development of national COVID-19 response strategies. Although not all of these are relevant because of different modes of transmission, we have highlighted those that remain relevant for the COVID-19 response. Most important are a strong decentralized coordination system, a sensitive surveillance system rooted in communities, risk communication, and community engagement. Technical innovations including IPC, decentralized treatment facilities, and strengthening of health services to avoid secondary crises are also critical.
